# 

**Published:** 1892-06-18

**Authors:** 


**Notices of Births, Deaths, and Marriages,** are inserted in this column at a charge of 2s. 6d. for an announcement not exceeding four lines.

### NOTICE TO CORRESPONDENTS.

All MS., Letters, Books for Review, and other matters intended for the Editor should be addressed The Editor, The Lodge, Porchester Square, London, W.

The Editor cannot undertake to return rejected M.S., even when accompanied by stamped directed envelope.

**Notice to Advertisers and Subscribers.**

All Advertisements, Orders for Copies of The Hospital, and Business Communications should be addressed to The Publisher, *not to the Editor*, The Hospital, 140, Strand, W.C.

The Cost of Subscription, post free, is as follows:—Per week, 2½d.; per quarter, 2s. 6d.; per half-year, 5s.; per annum, 8s. 6d. Foreign:—Per annum, 10s. 8d.; payable, in all cases, in advance.

**"Burdett's Hospital Annual," 1891—1892.**

Third year of publication. 600 pp. Boards, gilt lettering. A most complete guide to British and Colonial Hospitals, Dispensaries, Nursing and Convalescent Institutions, and Asylums. Price 3s. 6d. Published by The Scientific Press (Limited), 140, Strand, W.C.

**NOTICE.—The Index for Vol. XI. (ending March, 1892) is on sale at the Office of this Journal, price 2½d., post free.**

### SCRAPS AND GLEANINGS.

THE date of the Belfast Hospital Saturday has been altered from the 11th to the 18th of June.

THE total amount collected on Hospital Saturday for the Homœopathic Hospital, Bath, was £106 12s. 2d.

SIR SMITH CHILD has just promised £100 towards forming a supplementary convalescent fund for Longton.

THE Duchess of Portland opened a new wing at the East London Hospital for Children, on Saturday, 11th inst.

THE Queen has given a donation of £50 to the fund for the relief of the distress caused by the disastrous hurricane at Mauritius.

THE Duke of Fife presided over the 40th general meeting of the subscribers to the Hospital for Sick Children, Great Ormond Street.

DURING the past 12 months 64 patients were treated at the Lynton District Cottage Hospital. The expenditure exceeded the income by £5 10s. 3d.

MRS. JONES, wife of a retired farmer, of Rhewl, Ruthin, North Wales, has been killed by lightning. The flash crashed through the house gable, and killed her as she stood by the fire.

DONATIONS amounting to over £5,000 have been promised in answer to an appeal to carry out an extension to the Poplar Hospital for Accidents, which is situated close to the East and West India Docks.

A BRASS band contest and athletic sports are to take place on August 1st (bank holiday) in Wharton Park, the proceeds of which are to be given to the Convalescent Home for Friendly Societies at Grange-over-Sands.

IN Greater London last week—that is, the London which includes 5,752,204 inhabitants—the births registered were just one more than twice the number of deaths. The respective totals were 3,515 and 1,757.

WHEN the Merchant Seamen's Orphan Asylum was first started at Snaresbrook only 11 children could be received. Now 200 boys and 100 girls are happy, contented, and well trained within the walls of the institution.

THE Rector of Blairgowrie and Rattray writes that a cottage hospital is much and sorely needed in the district. He appeals specially to all who are strong in health, robust and vigorous, to contribute towards the erection of such a building.

PRINCESS CHRISTIAN has consented to lay the foundation stone of the New British Home for Incurables at Streatham on the afternoon of Saturday, July 16th, when invitations to a garden party will be issued by the President and Committee.

THE anniversary festival of the Royal Asylum of St. Anne's Society was held at Salters' Hall, St. Swithin's Lane, Mr. Samuel More Richards in the chair. Children of professional men belonging to any country are eligible for admission, between the ages of 7 and 12.

At the annual meeting of the Governors of the Devon and Exeter Hospital Sir John Shelley, President of the institution, stated that the annual accounts showed a deficit of nearly £1,000. The total number of in-patients was 1,456, and of out-patients 2,323.

THE Council of the London Diocesan Penitentiary, Highgate, are very anxious to raise £1,100 to complete the alterations in the laundry and to build new dormitories for 20 young women. The work has been in hand for 12 months, and is now stopped for want of funds.

AT a meeting convened by the Charity Organization Society which took place at the rooms of the Society of Arts, Mr. Leslie Stephen read an address on "Considerations upon the modern tendency to propose heroic remedies for social evils; its causes and bearings."

THE annual bazaar in connection with the Mauldeth Hospital for Incurables was held recently. Last year out of the fund formed in this way as much as 30s. was given to each patient, and £34 7s. was presented to them to defray their travelling expenses when visiting friends.

AT the the ninetieth annual meeting of the Tynemouth Dispensary, the Secretary stated that the charge of twopence made for each casual patient supplied with medicine during 1891 had realised £40 10s. 11d. The receipts amounted to £301 4s. 3d., and the ordinary expenditure to £275 14s. 4d.

A SCHOLARSHIP of £35 a year for three years will be offered for competition among women students at an examination to be held at Westfield College, Hampstead, early next month. The successful candidate will be required to work for one of the degrees of London University (B.A. or B.Sc.).

OWING to the munificence of Sir Donald Smith, the Forres Leanchoil Hospital is now an accomplished fact, and the buildings have been thrown open for patients. Mr. John Rhind, of Inverness, was the architect, but owing to his sudden death the work was carried out by Mr. Saxon Snell, of London.

UNDER the patronage of the Queen, the Prince and Princess of Wales, the Duke and Duchess of Connaught, Princess Christian, Princess Louise, Princess Beatrice, and Princess Henry of Battenberg, a morning concert will be given on June 27th, at St. James's Hall, in aid of the Society for the Prevention of Cruelty to Children.

THE third jubilee of the Royal Mineral Water Hospital, Bath, was celebrated by 165 patients, who were feasted with roast meat and plum pudding for dinner and in the evening with cake and other refreshments. The female patients amused themselves with singing and dancing whilst the male patients held a smoking concert.

ABOUT 150 ladies and gentlemen attended the dance at Westminster Town Hall in aid of the funds of the London Skin Hospital, Fitzroy Square. The Committee hope to make the place more of a home than a hospital, and that the Institution shall eventually become self-supporting. In furnishing the wards a debt of some £900 has been incurred.

A THREE days' bazaar in aid of the Christian Blind Relief Society was held recently in the Westminster Town Hall. The Princess of Wales, who is a Patron of the society, sent 72 small pieces of china for sale on the stalls. This society has about four hundred pensioners on its books who receive 10s. or £1 per month. The majority of them apart from the pension have not twopence a day.

### The Student of Medicine.

[All communications for this Supplement should be addressed to The Editor of The Hospital, at 140, Strand, with the words, "Students' Column," written in the left hand top corner of the envelope.]

### APPOINTMENTS.

Anderson, A. W., M.B., C.M.Edin., appointed Assistant Medical Officer to the Fife and Kinross District Asylum, Cupar. Arnison, W. C., M.D.Durh., M.R.C.S., appointed Professor of Surgery in the University of Durham. Biernacki, J., M.B., C.M., L.R.C.S., L.R.C.P.Edin., appointed Assistant House Surgeon to Bootle Borough Hospital. Butlin, Henry Trentham, F.R.C.S., appointed Surgeon to St. Thomas's Hospital. Clemmey, W.N., M.R.C.S.Eng., L.R.C.P.Lond., appointed House Surgeon to Bootle Borough Hospital. Couper David, M.D.Glas., appointed Surgeon for Diseases of the Skin to the Dispensary of the Victoria Infirmary, Glasgow. Fletcher, James L., M.B., C.M.Edin., appointed Resident Medical Officer to the Manchester Hospital for Consumption and Diseases of the Throat. Goodall, E. W., M.D.Lond., appointed Medical Superintendent of the Eastern Fever Hospital. Hirst, Walter, L.R.C.P., L.R.C.S.Edin., appointed Resident Assistant Medical Officer at the Leeds Union Infirmary. Hunter, George, L.F.P.S.Glas., appointed Surgeon for Diseases of the Eye to the Dispensary of the Victoria Infirmary, Glasgow. Jenkins, Thomas W., M.A., M.D.Glas., appointed Physician to the Victoria Infirmary Dispensary, Glasgow. Kelly, A. Brown, B.Sc., M.B., C.M.Glas., appointed Surgeon for Diseases of the Throat, Ear, and Nose to the Dispensary of the Victoria Infirmary, Glasgow. Wyman, C., M.A., M.B., B.C.Cantab., L.R.C.P., M.R.C.S., reappointed Resident House Physician to St. Thomas's Hospital.

### VACANCIES.

The following vacancies are announced this week, the amount of salary in each case being given after the name of the institution:

#### Resident Medical Officers.

Brighton and Hove Lying-in Institution and Hospital for Women, 76, West Street, Brighton—House Surgeon.

Chorlton-upon-Medlock Dispensary, Manchester—Resident House Surgeon; doubly qualified. Salary, £100 per annum.

Derby Borough Asylum, Rowditch, Derby, Assistant Medical Officer—Salary, £100 per annum, with board and washing.

Dundee Royal Lunatic Asylum, Clinical Assistant.

General Hospital, Birmingham, Assistant House Surgeon—Board, &c.

General Hospital, Birmingham, Resident Medical Officer; doubly qualified—Salary, £130 per annum, with board, &c.

Sheffield Public Hospital and Dispensary, Assistant House Surgeon—Salary, £50 per annum, with board, &c.

Warneford Hospital, Leamington, Spa, House Surgeon—Salary, £100 per annum, with board, &c.

Wolverhampton and Staffordshire General Hospital, Wolverhampton, House Physician—Salary, £100 per annum, with board, &c.

#### Non-Resident Medical Officers.

Bradford Eye and Ear Hospital, Hallfield Road, Bradford, Special Assistant Surgeon—Honorarium, 100 guineas per annum. Must reside near the hospital.
Dental Hospital of London and London School of Dental Surgery, Leicester Square, Demonstrator—Honorarium £50 per annum.
Evelina Hospital for Sick Children, Southwark Bridge Road, Physician to Out-patients.
Gainsborough Friendly Societies'. Medical Association, Resident Medical Officer, not under 28 years of age—Salary, £200 per annum, together with midwifery fees, house rent, and rates.
Manchester and Salford Provident Dispensaries' Association, vacancy on the Medical Staff of the Ancoats Branch.
Manchester Hospital for Consumption and Diseases of the Throat and Chest, Hardman Road, Deansgate, Manchester, Honorary Assistant Physician.
Mason College, Birmingham, with Queen's Faculty of Medicine, Professorship of Medicine, Lectureship on Ophthalmology, Lectureship on Dental Surgery and Dental Pathology.
Royal Westminster Ophthalmic Hospital, King William Street, West Strand, Clinical Assistants—Appointments for six months.
Westminster General Dispensary, 9, Gerrard Street, Soho, W., Honorary Physician.
Westminster Hospital, Broadway Sanctuary, S.W., Assistant Ophthalmic Surgeon; must be F.R.C.S.Eng.
Wolverhampton and District Hospital for Women, two Honorary Acting Gynæcological Surgeons.

### ATHLETICS.

#### Charing Cross Hospital.

Delightful weather favoured the annual athletic sports held by the students attached to Charing Cross Hospital last week, but, notwithstanding, the attendance at Stamford Bridge Grounds, Fulham Road, was by no means up to the average for an event of this kind. As usual the programme embraced the customary contests: 100 yards sprint,

### THE STUDENT OF MEDICINE—(*continued*).

long jump, 120 yards hurdles, half-mile handicap, putting the shot, half-mile handicap open to other metropolitan hospitals, one mile bicycle race, 440 yards, one mile flat race, and throwing the cricket ball. The best form was shown by A. R. McCullagh, who carried off the half-mile from scratch, the quarter, and the one mile, penalised 15 yards. J. H. Fegan won the 100 yards and long jump, whilst, with 20 yards start, M. E. Tressider (Guy's Hospital) did 2 min. 2 3-5 sec. in the open half-mile. The officials were: Judges, Mr. H. F. Waterhouse, F.R.C.S., and Mr. F. Willcocks, M.D.; referee, Mr. J. A. Bloxham, F.R.C.S.; starter, Mr. C. J. Woollett, F.R.C.S.; timekeeper, Nat Perry (L.A.C.). The band of the 2nd Middlesex Rifles played a nice selection of music, and Mr. H. Green presented the prizes. Details:

100 Yards Scratch Race.—First heat: J. H. C. Fegan, 1; W. H. Thomas, 2; A. E. Wild, 0; W. E. Wyborn, 0; W. Mudie, 0; S. L. Steggall, 0. Won by a yard. Time, 11 1 5 sec. Second heat: H. T, Roberts (pen. 1 yard), 1; E. S. Lovell-Lovell, 2; M. Carter, 0; W. H. Morgan, 0; R. F. Wallace, 0; M Molloy, 0; A. Mudie, 0. Won by a yard. Time, 11 1-5 sec. Final heat: Fegan, 1; Roberts, 2; Lovell-Lovell, 3; Thomas 0. Good race; won by three-quarters of a yard; half a yard between second and third.

Long Jump.—J. H. Fegan, 18 ft. 4½ in.; A. Mudie, 16 ft. 4¼ in.; M. Carter, L. F. Forbes, Winslow, M. Molloy, W. H. Morgan, and H. Gardner also competed.

120 Yards Hurdles.—S. L. Steggall (pen. 2½ yards), 1; J. H. Fegan, 2; L. Hammond, 0; C. Cock, 0; A. Mudie, 0; H. Gardner, 0; Lovell-Lovell, 0. Won by 2 yards. Time, 20 1-5 sec.

Half-mile Handicap.—A. R. McCullagh, scratch, 1; F. A. Lucas-Hammond, 35 yards start, 2; A. Mudie, 10, 0; M. Molloy, 30, 0; P. A. Longhurst, 35, 0; H. H. Woods, 55, 0; A. E. Walker, 60, 0. The limit man maintained the command for a lap and a bit, but he was then done with, and McCullagh securing the lead 230 yards from home, came right away, and won by 20 yards, running throughout in capital form. Time, 2min. 7 4-5 sec.

Putting 16 lb. Shot.—M. Molloy (pen. 1 ft.), 32 ft. 10 in; F. A. Lucas-Hammond, 28 ft. 5 in; H. Thomas, 27 ft. 9 in. Six others competed.

Open-half Mile Handicap.—M. E. Tressider (Guy's), 20 yards start, 1; H. W. Dudgeon (Guy's), 30, 2; E. C. Clements (University), 40, 0. Splendid race all through, the winner just struggling home by a yard. Time, 2 min. 2 3-5 sec.

One Mile Bicycle Race.—P. C. Spark, scratch, 1; H. A. Madge, 40 yards start, 2; A. Mudie, 140, 0. The scratch man took the lead commencing the last lap, and won easily by 50 yards. Time, 3 min. 11 2-5 sec.

440 Yards Scratch Race.—A. R. McCullagh, 1; H. T. Roberts (pen. 5 yards), 2; J. H. Fegan, 0; H. H. Thomas, 0; Lovell-Lovell, 0. Roberts took the lead at half-distance, but McCullagh came again, and the two were dead level down the straight. The latter stayed the longer, however, and Roberts tiring, M[illegible]h drew away and won by eight yards. Time, 55 sec.

Throwing the Cricket Ball.—M. Molloy, 81 yards 2 ft. 8 in.; J. H. Fegan, 81 yards 2 ft.; C. W. Young, N. S. Bickford, L. Newton, E. C. Montgomery, Yorke Davies, P. Phillips, A. Mudie, Lovell-Lovell also competed.

One Mile Flat Race.—A. R. McCullagh (pen. 15 yards), 1; A. E. Weld, †; P. A. Longhurst, †; A. Mudie (pen. 5 yards), 0; Lucas-Hammond, 0; E. V. Fooks, 0. McCullagh was in front before the completion of the first lap, and he eventually won easily by 40 yards from the dead-heaters, who were together all the way. Time, 5 min. 10 2-5 sec.

Tug of War and 220 Yards Consolation Race brought the proceedings to a close.

### Westminster Hospital.

The third annual athletic meeting of the students and others connected with Westminster Hospital took place last week at Stamford Bridge. G. C. Hancock was particularly successful in the closed events. E. H. Pelling's fine performance in the 300 Yards Open Handicap was the feature of the meeting. The leading officials were as follows:—Referee, Mr. W. H. Allchin, M.B., F.R.C.P.; starter, Mr. G. Fletcher, M.D.; timekeepers, Dr. Fitzgerald Arthur and Nat Perry; judges, Drs. J. Black, F.R.C.S., and H. B. Donkin, M.B.; hon. treasurer, Mr. P. Pierre; hon. sec., Mr. Henry Morris. Results:—

100 Yards Handicap.—G. C. Hancock, scratch, 1; A. E. Rutherford, 7 yards start, 2. Won by a foot. Time, 11 sec.

Putting the Weight.—R. Pierre, 31 ft. 8½ in., 1; S. B. Blomfield 29 ft. 0 in., 2.

Half-mile Handicap.—R. Nitch Smith, 40 yards start, 1; G. C. Hancock, scratch, 2. Won by 20 yards. Time, 2 min. 13 sec.

High Jump.—A. H. M. Hood, 4 ft. 10½ in., 1; P. Pierre, who was penalised 9 in., retired on reaching 4 ft. 11½ in.

One Mile Handicap.—H. R. Tourne, 120 yards start, 1; R. A. Macleod, scratch, 2. Won by 80 yards. Time, 5 min. 3 4-5 sec.

Quarter of a Mile Handic[illegible]—S. B. Blomfield, 20 yards start, 1; A. J. Rutherford, 30, 2; G. C[illegible]cock, scratch, 3. Two yards covered the three. Time, 54 1-5.

Long Jump.—G. C Hancock, 20 ft. 0½ in., 1; S. R. Walker, 16 ft. 11 in., 2.

100 Yards Hurdles.—J. M. Swainson, 1; P. Pierre, 2. Won by a foot. Time, 20 2-5 sec.

300 Yards Invitation Handicap.—E. H. Pelling, L.A.C., scratch, 1; A. Ovenden, L.A.C., 7 yards start, 2; W. Lotinga, L.A.C., 34, 3. Lottinga led until well into the straight, when he was passed by Ovenden. The latter was beaten on the post by Pelling by a few inches, with Lottinga a yard behind. Time, 31 4-5 sec.

An Obstacle Race and Tug of War were included in the programme.

